# Recurrent Gastric Gastrointestinal Stromal Tumor in a Patient with Neurofibromatosis

**DOI:** 10.7759/cureus.2854

**Published:** 2018-06-21

**Authors:** Laith A Al Momani, Omar Abughanimeh, Lindsey C Shipley, Jennifer Phemister, James Swenson, Mark Young

**Affiliations:** 1 Department of Internal Medicine, East Tennessee State University, Johnson City, USA; 2 Department of Internal Medicine, University of Missouri Kansas City School of Medicine, Kansas City, USA; 3 Department of Internal Medicine, University of Alabama, Birmingham, USA; 4 Department of Gastroenterology, East Tennessee State University, Johnson City, USA; 5 Department of Gastroenterology, Mountain Home Veterans Affairs Hospital, Mountain Home, USA

**Keywords:** gastrointestinal bleed, gists, iron deficiency anemia

## Abstract

Neurofibromatosis type 1 is an autosomal dominant neurocutaneous disorder characterized by a mutation of the neurofibromin 1 (NF1) gene, resulting in increased susceptibility for multiple tumors, namely, gastrointestinal stromal tumors (GISTs)—the most common types of mesenchymal neoplasms in the gastrointestinal tract. Despite these tumors' predilection for the stomach, it seems to be the least likely part of the gastrointestinal (GI) tract to be affected in cases of neurofibromatosis. Herein, we report a case of a 61-year-old male patient with known neurofibromatosis, who presented with acute blood loss anemia due to a recurrent gastric GIST, requiring partial gastrectomy due to its size and multiple recurrences.

## Introduction

Neurofibromatosis type 1 (NF1) is an autosomal dominant genetic disorder with a gene mutation of neurofibromin 1, which has been found to increase susceptibility to multiple types of tumors, namely, gastrointestinal stromal tumors (GISTs) [1]. In addition to GISTs, these patients have an increased incidence of neurofibromas, malignant peripheral nerve sheath tumors (MPNST), and gliomas [2]. GISTs are the most common soft tissue sarcomas arising in the gastrointestinal (GI) tract [3]. Here, we have a patient with neurofibromatosis who presented with blood loss anemia due to a bleeding, recurrent gastric GIST.

## Case presentation

A 61-year-old male patient with a known diagnosis of neurofibromatosis type I presented to the emergency department with a complaint of melena of two days duration. He had been complaining of fatigue and lightheadedness as well. He denied any nausea, vomiting, or abdominal pain. The use of nonsteroidal anti-inflammatory drugs was denied.

His past medical history is significant for an asymptomatic GIST on esophagogastroduodenoscopy (EGD) screening that was treated with neoadjuvant imatinib therapy and, subsequently, completely resected three months prior to presentation.

The physical examination revealed no abnormal findings. Laboratory testing was remarkable for blood urea nitrogen (BUN) 37 mg/dL, creatinine 1.1 mg/dL, hemoglobin (HB) 6.5 g/dL, and mean corpuscular volume (MCV) 78.7 fL/red cell.

**Figure 1:**
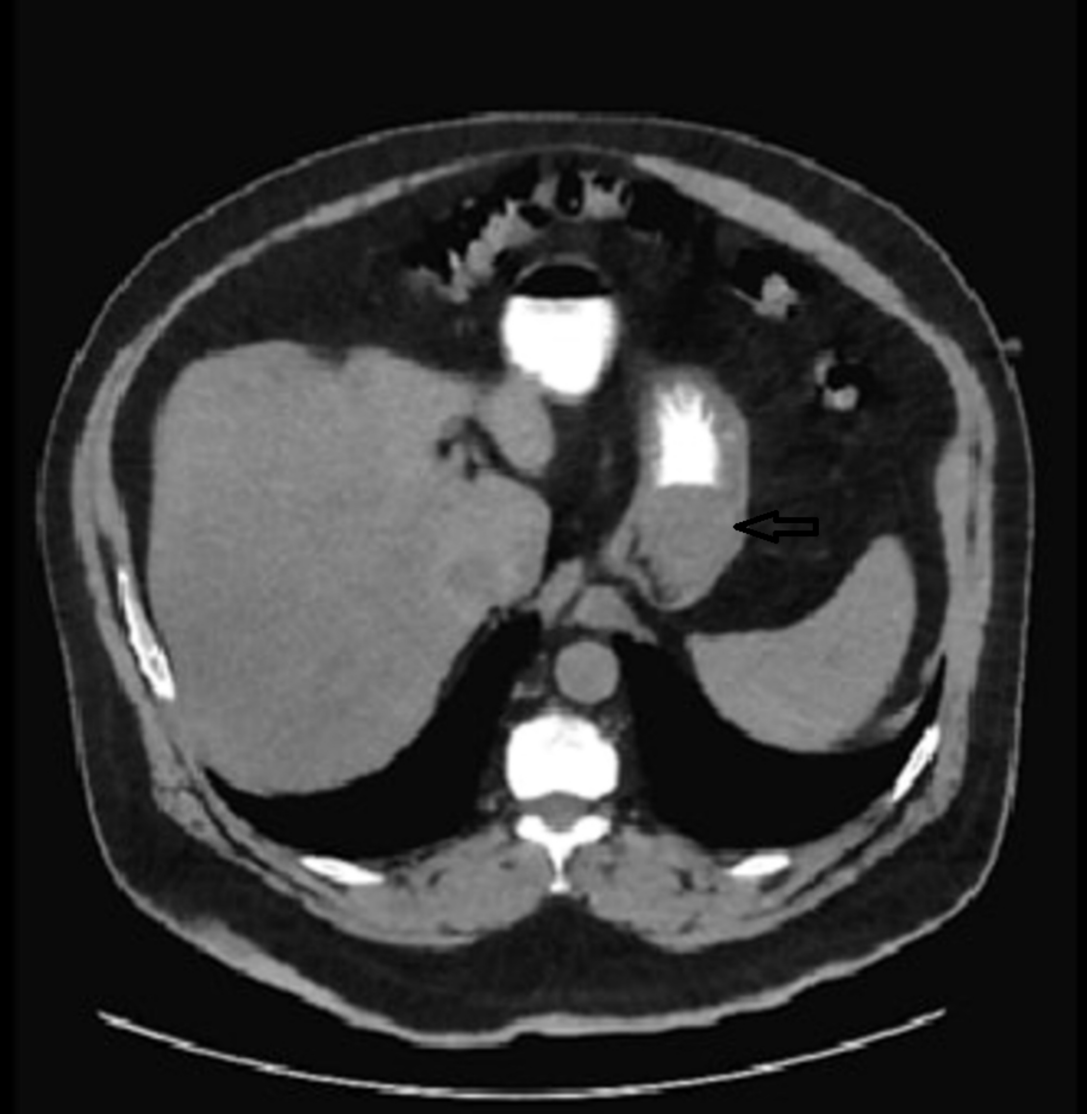
CT scan of the abdomen - transverse view showing a suspected mass in the stomach CT: computed tomography

A computed tomography (CT) scan of the abdomen (Figure 1) was performed and elicited a suspected mass in the stomach. No signs of metastasis were present.

The patient was admitted and gastroenterology was consulted.

**Figure 2:**
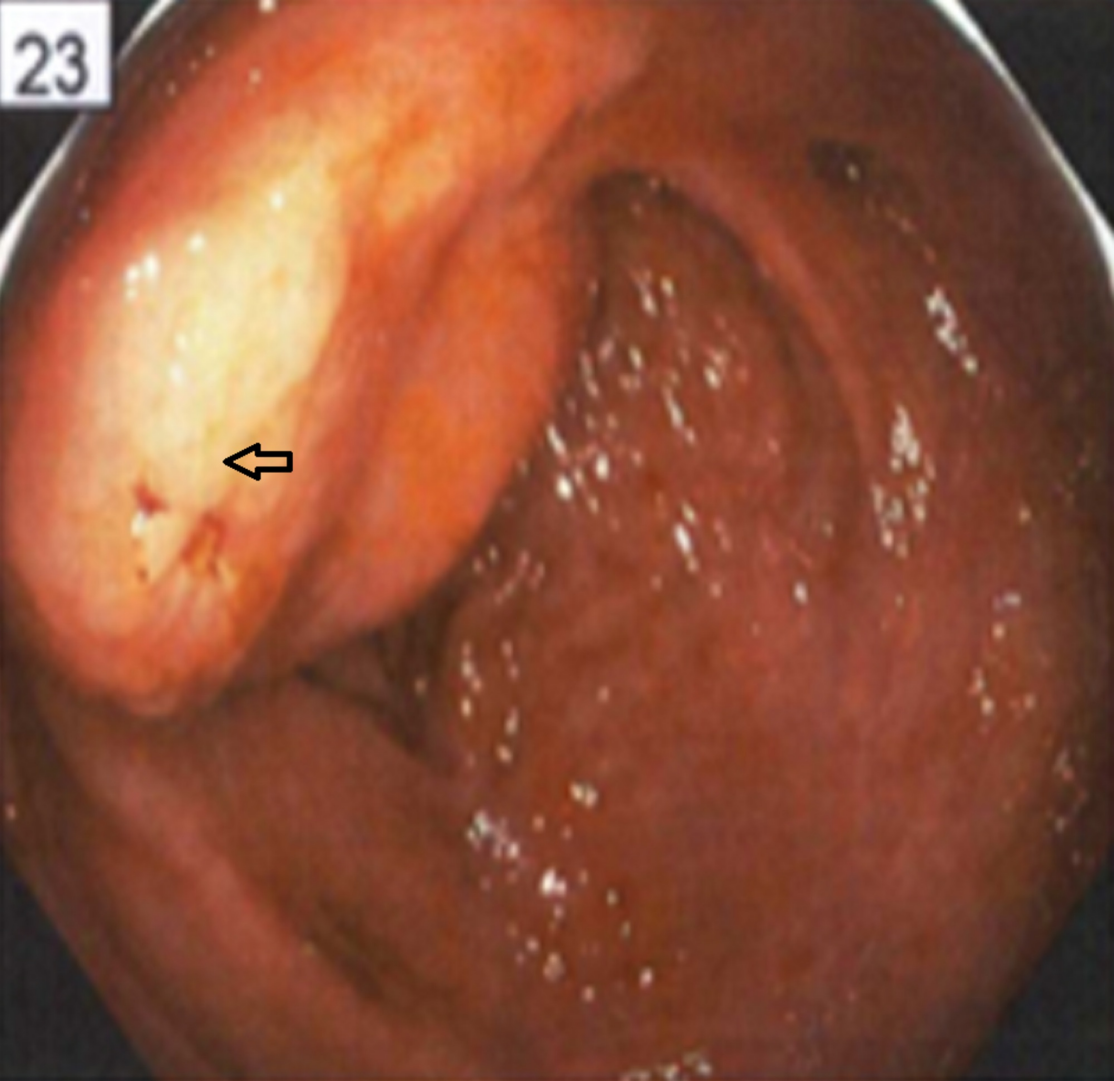
A 5-cm gastric mass in the proximal posterior body of the stomach with bleeding stigmata

He underwent an EGD (Figure 2), which showed a 5-cm gastric mass in the proximal posterior body of the stomach with bleeding stigmata.

**Figure 3:**
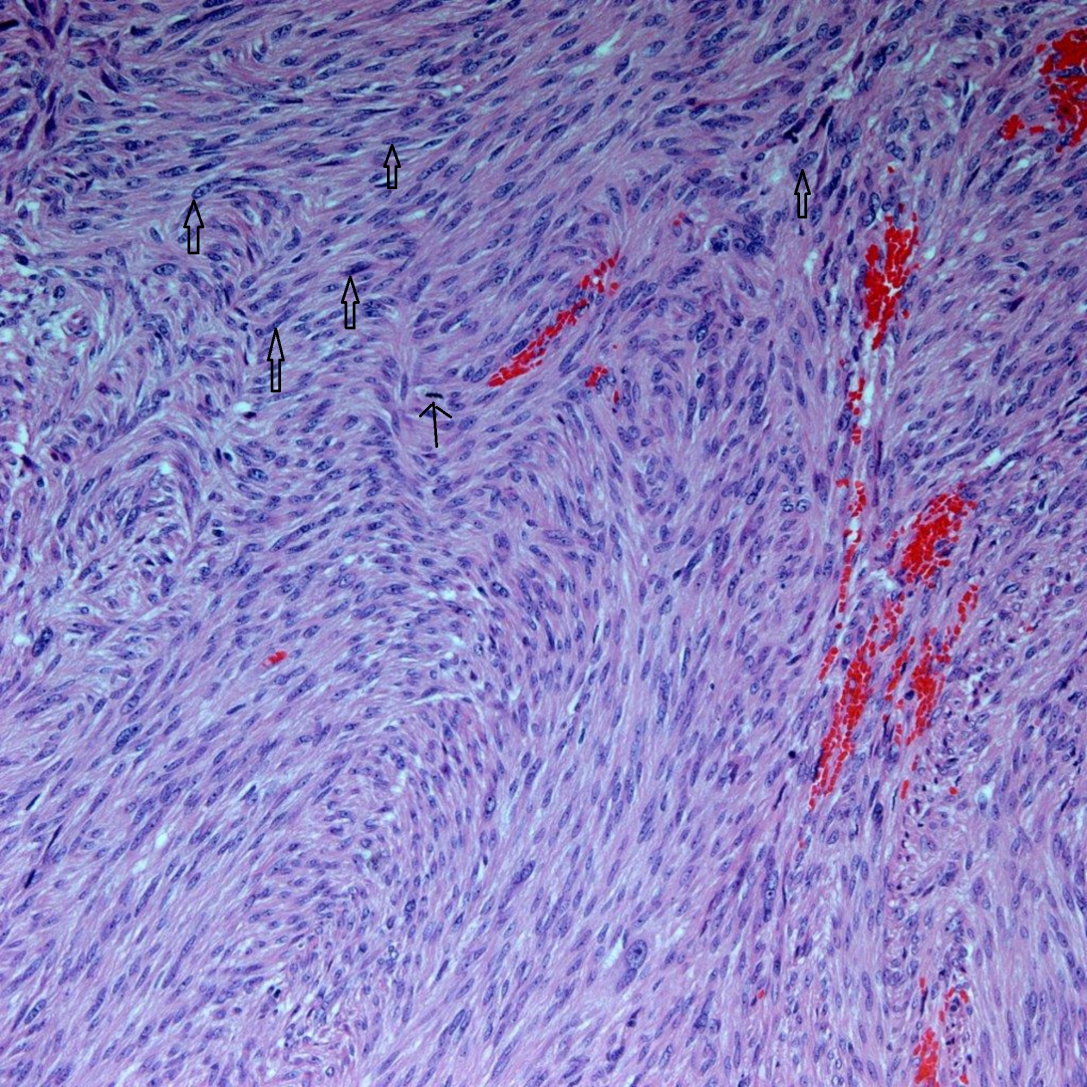
Gastric GIST, spindle cell (x200) Thick arrows: examples of neoplastic cells; thin arrow: mitotic figure

**Figure 4:**
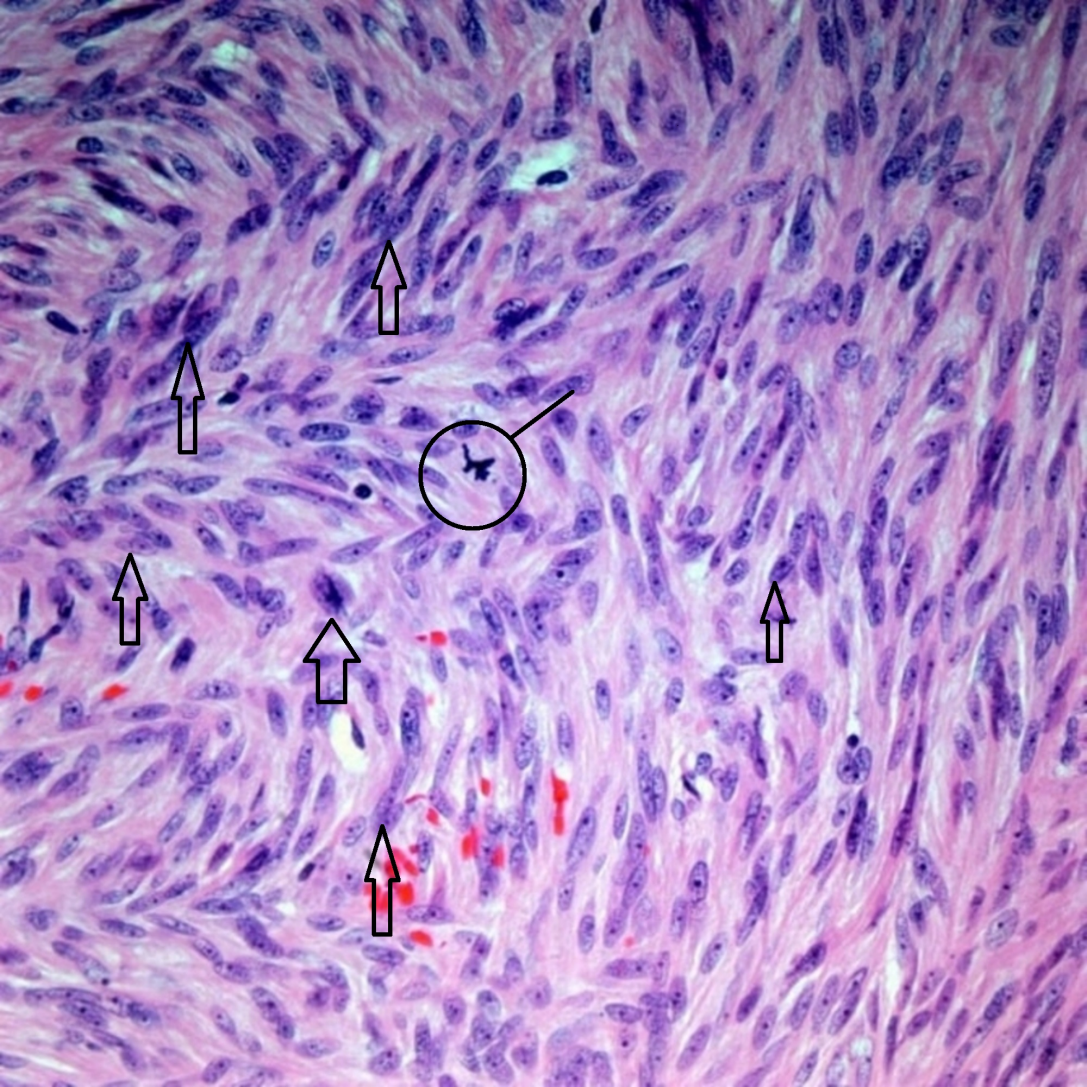
Gastric GIST, intermediate risk with >5 mitosis/50hpf (x400) Thick arrows: examples of neoplastic cells; circle: atypical mitotic figure GIST: gastrointestinal stromal tumor

**Figure 5:**
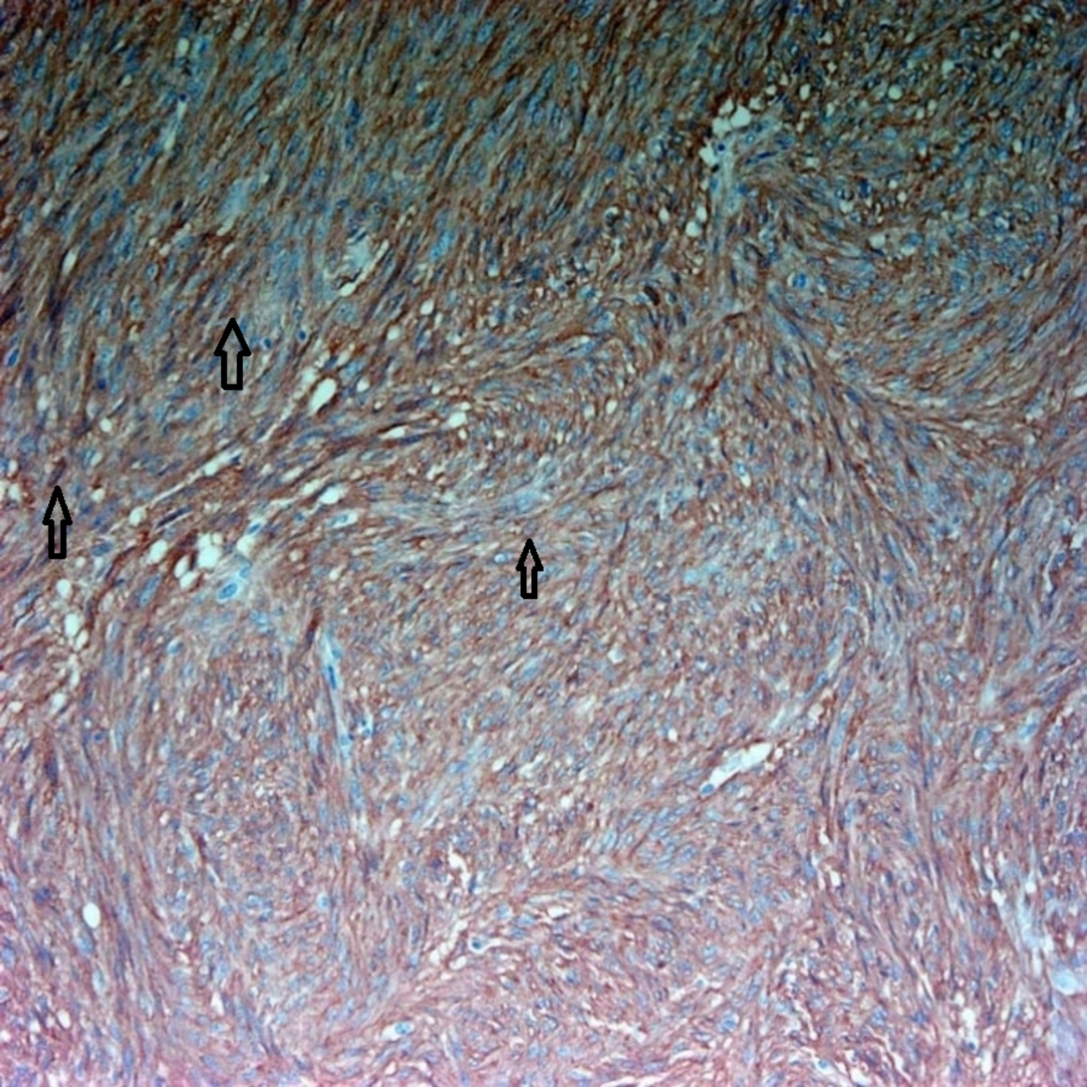
Tumor cells are immunoreactive for CD117 A CD117 (c-Kit) immunohistochemical stain showing diffuse cytoplasmic positivity, which is typical for GIST (the brown color is the positivity and it fills the cytoplasm of the neoplastic cells and spares the nuclei). GIST: gastrointestinal stromal tumor

The pathology report was consistent with GIST, as can be seen in Figures 3-5.

The patient underwent laparoscopic partial gastrectomy without complication, given the size of the GIST, and recurrence following a consultation with the surgery department.

## Discussion

NF1 is an autosomal dominant disorder with a gene mutation of neurofibromin 1, which has been found to increase susceptibility to multiple types of tumors, namely GISTs [1,4].

Although GISTs are quite rare, they are the most common soft tissue sarcomas of the GI tract. Most studies report an incidence of 10-15 per million per year. The most common location is gastric (55%) followed by small bowel (31.8%), colon (6%), other/various locations (5.5%), and esophagus (0.7%) [5]. On the other hand, GISTs in patients with neurofibromatosis are most commonly found in the small intestine and are rarely seen in the stomach, making this case even more unique [1,5].

The average age of diagnosis is 60 years with no gender specificity. However, there is a higher incidence in those living in China, Taiwan, and Norway with an incidence of 19-22 per million per year [5].

GISTs arise from the cells of Cajal within the mesenchymal tissue of the gastrointestinal tract [3,6]. Approximately 80% of GISTs have KIT gene mutations that lead to activation of KIT receptors, which plays an important role in tumorigenesis [7].

Clinical features vary depending on the location, size, and aggressiveness of the tumor with gastrointestinal bleeding being the most common presentation. However, GISTs are frequently found incidentally [8-10].

Appropriate diagnostic workup with EGD and/or endoscopic ultrasound (EUS), as well as imaging with abdominal CT or magnetic resonance imaging (MRI), is warranted [11].

Treatment options for patients with both neurofibromatosis type I and GISTs include surgical resection when possible. In addition, there has been a very good response to adjuvant and neoadjuvant imatinib, a tyrosine kinase inhibitor, given the frequent presence of kinase mutations in these tumors [2]. The risk of recurrence and mortality is very similar between NF1 and non-NF1 patients following surgical resection [4].

Of note, there have been 11 reported cases of GISTs and pheochromocytomas in patients with NF1. Therefore, these patients should be screened for this third entity prior to undergoing any surgical resection, as it carries a high perioperative cardiovascular mortality risk if untreated [1].

This case was presented in abstract form as a poster at the World Congress of Gastroenterology in October 2017:

Recurrent GIST Tumor in a Patient with Neurofibromatosis; Al Momani L, Crosnoe-Shipley L, Phemister J, Swenson JA, Sigei A, Velilla R, Young M; American Journal of Gastroenterology 2017, volume 112, issue S1, 2533.

## Conclusions

Known NF1 patients with vague abdominal symptoms or signs suggestive of gastrointestinal bleeding, including iron deficiency anemia, should be screened for GISTs given the imperative association between these two uncommon entities. Therefore, early detection and, hence, early intervention is imperative in these patients in order to improve survival and quality of life.
